# Iron in the Blood

**Published:** 1913-11

**Authors:** 


Iron in the Blood. P. H. C. Fowell, *Quart. Jour. of Med.*, 1912, page 179, states that normally, on the average, about a quarter of the iron in the blood is not combined as hæmoglobin, and that, in pernicious anæmia, half the total iron may be uncombined. This seems to be the most valuable hint as to the real nature of pernicious anæmia that has been given for a long time. Superficially, we may consider pernicious anæmia to be a specific failure of assimilation of iron by the red cells, and not a gross lack of iron. The next and more important, and probably more difficult question is why do not the red cells assimilate the available iron into hæmoglobin?

